# Field Trials of an Autonomous eDNA Sampler in Lotic Waters

**DOI:** 10.1021/acs.est.4c04970

**Published:** 2024-11-14

**Authors:** Scott D. George, Adam J. Sepulveda, Patrick R. Hutchins, David S. Pilliod, Katy E. Klymus, Austen C. Thomas, Ben C. Augustine, Chany C. Huddleston Adrianza, Devin N. Jones, Jacob R. Williams, Eric G. Leinonen

**Affiliations:** † U.S. Geological Survey, New York Water Science Center, Troy, New York 12180, United States; ‡ U.S. Geological Survey, Northern Rocky Mountain Science Center, Bozeman, Montana 59715, United States; § U.S. Geological Survey, Forest and Rangeland Ecosystem Science Center, Boise, Idaho 83702, United States; ∥ U.S. Geological Survey, Columbia Environmental Research Center, Columbia, Missouri 65201, United States; ⊥ Smith-Root, Vancouver, Washington 98686, United States; # U.S. Geological Survey, Eastern Ecological Science Center, Laurel, Maryland 20708, United States; ∇ Turner Institute of Ecoagriculture, Natural Resources Program, Bozeman, Montana 59718, United States

**Keywords:** robotic sampler, temporal, invasive, Rainbow Trout, Round Goby, Spectaclecase, Western Pearlshell, Westslope Cutthroat Trout

## Abstract

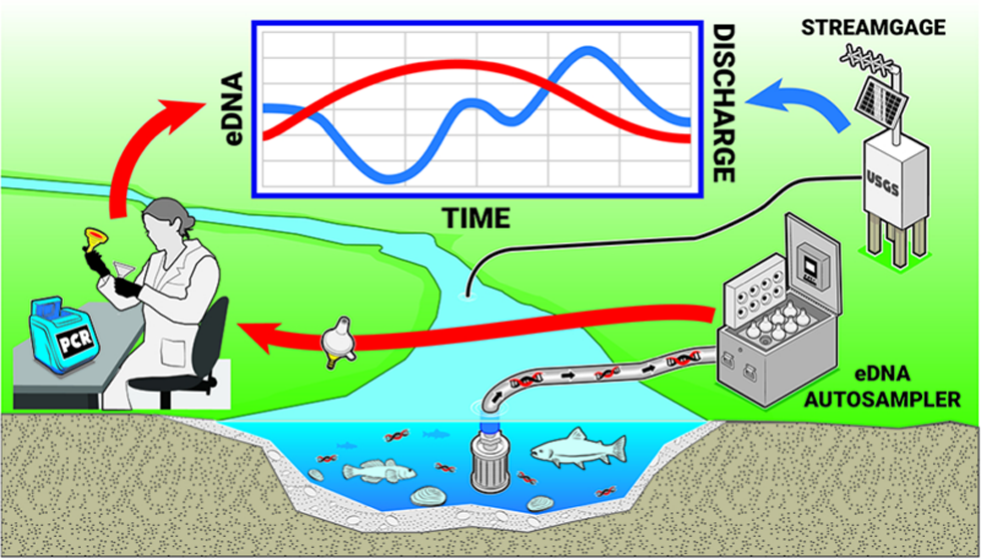

Environmental DNA (eDNA) analysis has become a transformative technology, but sample collection methods lack standardization and sampling at effective frequencies requires considerable field effort. Autonomous eDNA samplers that can sample water at high frequencies offer potential solutions to these problems. We present results from four case studies using a prototype autonomous eDNA sampler as part of the U.S. Geological Survey’s Rapid Environmental eDNA Assessment and Deployment Initiative & Network (READI-Net) project. These case studies involved short-term deployments of an eDNA autosampler (Smith-Root) across a range of riverine habitats with the objectives of (a) identifying what insights could be gained from high-frequency autosampling and (b) benchmarking these autosamples against manually collected samples. The high frequency autosampling revealed high temporal variability of eDNA concentrations and provided valuable insights about eDNA associations with environmental covariates, such as discharge and turbidity. Benchmarking assessments indicated autosamples had similar detection rates to manual samples and obtained similar or greater eDNA quantities. We did find minimal carryover contamination in autosampler field controls. We conclude that eDNA autosamplers have potential to improve freshwater biosurveillance by reducing logistical sampling barriers, standardizing collection methods, and clarifying the influence of environmental covariates on eDNA results.

## Abstract 2

We conducted a multiriver assessment of a prototype autonomous environmental DNA sampler and found this novel technology provides a solution to monitor biodiversity shifts that are occurring too rapidly for traditional tools to track.

## Introduction

Environmental DNA (eDNA) sampling is widely touted as having the potential to transform the fields of ecology and natural resource management.^1^ Contemporary occurrence of species inferred from eDNA samples is beginning to be used to inform management of native and invasive species.^2−4^ Despite widespread excitement, the application of eDNA methods is still hampered by a lack of standardization and consistent protocols.^1,5^ Additionally, temporal and spatial variation in eDNA concentrations in natural waters can limit the inference that can be made from single-visit or low-frequency eDNA samples.^6^

Autonomous eDNA samplers (hereafter “autosamplers”) are one potential solution to these problems because they can eliminate inconsistency or error associated with manually collected samples, standardize field methods, and enable high-frequency sampling. Autosampler platforms have been in development for 30 years in marine environments,^7^ but only a limited number of freshwater field trials have been conducted. These initial freshwater trials found that autosamplers had potential to provide long-term, high frequency data that were comparable to results from manual eDNA samples but the existing design was impractical for scalable implementation.^8−10^

Here we report on a new prototype robotic autosampler that can be operated by nonexpert users. Tests of this prototype were part of the U.S. Geological Survey’s (USGS) Rapid Environmental eDNA Assessment and Deployment Initiative & Network (READI-Net) project.^11^ Our goal was to assess the utility of autosampling technology in a variety of lotic freshwater environments. Specifically, we sought to (a) identify what additional insights could be gained from high-frequency eDNA sampling enabled by an autosampler and (b) benchmark the efficacy of an autosampler against established methods of eDNA sample collection. To achieve these objectives, we conducted four case studies during 2023 using an eDNA autosampler to sample for fish and mollusk eDNA targets across a diverse set of lotic habitats in the contiguous United States, ranging from a headwater stream in the Western region to a large river in the Northeastern region (Table 1). Each case study adhered to a number of consistent themes but also included adaptations to address specific local questions and challenges. Individually, these case studies demonstrate the flexible applications of this technology and provide useful insight about specific applications of the eDNA autosampler. Considered together, these case studies demonstrate that autosamplers have considerable potential to improve biomonitoring at temporal scales that are prohibitive to achieve with manual sampling. As with any method, however, there are trade-offs that must be considered relative to the study objectives and budget.

**Table 1:** Site Characteristics and Summary of Four Case Studies Conducted in 2023 Using an eDNA Autosampler

location	coordinates (dd)	elevation (m)	discharge (m^3^/s)	dates	sampling regime	qPCR targets	autonomous samples/field controls	measured covariates	manual benchmarking methods
Hudson River, NY	42.75226, −73.68908	5	328.84^a^	Jun 29-Jul 6, 2023	8 per day, every 3 h	Round Goby	56/2	discharge, turbidity	n/a
				Jul 6, 2023	method triplicates		8/2		backpack sampler, hand pump
Cherry Creek, MT	45.52284, −111.44619	1717	0.20^b^	Aug 14–21, 2023	8 per day, every 3 h	Westslope Cutthroat Trout	56/1	water level	grab samples for peristaltic pump
Loggers Creek, ID	43.58740, −116.17304	830	0.67^c^	Jul 26–28, 2023 & Aug 1–3, 2023	8 per day, every 3 h	Rainbow Trout, Western Pearlshell	32/1	discharge	backpack sampler
Big Piney River, MO	37.81587, −92.06985	230	5.75^d^	Sep 26, 2023	every 15 m for 4 h	Lake Trout, Spectaclecase	16/0	n/a	grab samples for centrifuge

a Mean daily average from June 29–July 6, 2023 at USGS 01358000 Hudson River at Green Island NY.

b Mean of three measurements taken between August 14–21, 2023.

c Mean daily average from July 26–August 3, 2023 at City of Boise, ID gage.

d Daily average from September 26, 2023 at USGS 06930060 Big Piney below Fort Leonard Wood, MO.

## Materials and Methods

### System Description and Setup

The eDNA autosampler (Smith-Root, Vancouver, WA) functions by suctioning water through self-preserving (desiccating) filter housings on a user-defined schedule. Broadly, the system components include (in flow order): an intake strainer, an intake hose, an upper manifold, a lower manifold, a pressure sensor, a diaphragm water pump in suction orientation, a flow sensor, and an outlet hose. Filters are placed between the upper and lower manifolds, creating independent sealed chambers for each filter with a pair of inlet and outlet valves that direct flow in and out of each chamber. The suction pump produces up to 12 -pounds per square inch (psi) for filtration in the sealed chambers. The eight filter locations are indexed and can be scheduled independently to filter at specified times, using the onboard software interface.

Each filtration event has three main phases: (1) System flush—water is suction pumped through the intake line (bypassing the filter manifold) and purged through the outlet to remove any residual water from a previous event, (2) Filtration—the inlet and outlet valves for a specified filter are opened and water is suctioned through the filter membrane until the target volume is reached or the minimum flow rate is detected, and (3) Air dry—an air valve is opened for one minute while the pump is running to convection-dry the filter membrane and remove any remaining water in the filter chamber and manifold. Once completed, the system goes into a low-power state while it awaits the next sampling event.

In all four case studies, the autosampler was installed on land and powered by a 12.8 V, 22.5 A-hour lithium iron phosphate rechargeable battery. At the start of each experiment, sterilized 6.35 mm ID polyurethane tubing was anchored to the ground and was not resterilized or replaced between samples. The water intake screen (250-μm mesh) was affixed to the terminal end of the tubing and mounted on a vertical support positioned in the water column.

The autosampler was programmed to filter 2 L of water with a target flow rate of 1.0 L/min and would discontinue pumping sooner if the flow rate dropped below 0.3 L/min (indicating filter clogging). A 5-L flush of the entire system was conducted using ambient river water immediately prior to collection of each sample. We were particularly interested in screening for carryover contamination between samples resulting from reusing the permanent water intake lines. Field controls (i.e., blanks) were taken periodically by manually removing the intake filter from the water, immersing it in a sterilized bucket filled with deionized water, conducting the standard 5-L flush, and then collecting a 2-L sample.

All samples taken by the autosampler were collected on single-use Smith-Root self-preserving (desiccating) 5-μm polyethersulfone (PES) filters.^12^ In all four case studies, paired (i.e., same place and time) manual eDNA sampling was conducted to benchmark the autosampler against previously established sample collection methods in the respective study areas. Further details unique to each deployment are described in the section below.

### Case Study #1: Hudson River, Albany NY

**Figure 1:**
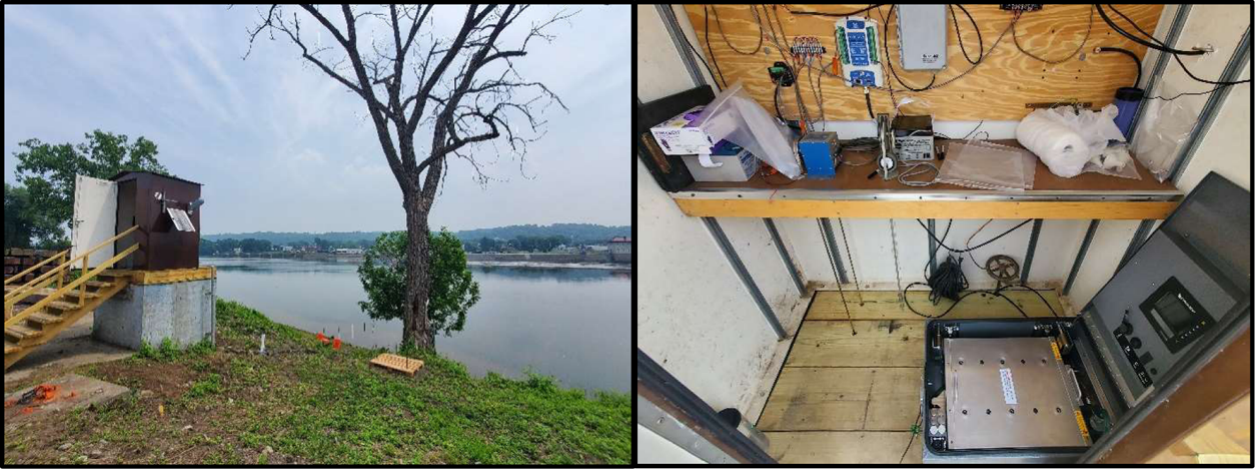
Deployment of the eDNA autosampler in a USGS streamgage on the Hudson River.

The autosampler was installed in a USGS streamgage (Figure 1) on the Hudson River approximately 13 km north of Albany, NY.^13^ The streamgage is located near river kilometer 248 and is approximately 0.2 km upstream of the Troy Dam (and associated lock and hydroelectric facility), which is the first barrier moving upstream from the Atlantic Ocean. At this location, the river has an approximate elevation of 5 m above sea level, width of 280 m, drainage area of 20,953 km^2^, and average annual discharge of 411.7 m^3^ per second (m^3^/s).^13^ The water intake was located approximately 5 m vertically (lower) and 17 m horizontally from the gage house, requiring the autosampler to suction water across this rise and run. In addition to providing protection for the autosampler, the gage recorded instantaneous (15 min interval) river stage, velocity, and turbidity data during the deployment. Discharge was computed using the index velocity method^14^ and turbidity was monitored using a YSI EXO2 sonde and turbidity sensor (YSI Inc., Yellow Spring, Ohio).

We ran two experiments as part of this deployment. In the first, the autosampler was programmed to collect eight samples per day (one every three hours) for seven consecutive days from June 29 to July 6, 2023. In the second experiment, eight samples were collected in triplicate on a single day to compare DNA detection rates and concentrations ascertained from (a) the autosampler, (b) an eDNA backpack sampler (Smith-Root, Vancouver, WA) using the same 5-μm self-preserving PES filters, and (c) a manual handpump using 1.5-μm glass-fiber filters with freezing as the preservation method. The manual handpump collection method has been the standard eDNA monitoring approach in this system and is described in George et al.^15^ We collected two field controls with the autosampler during the first experiment, and two simultaneous field controls using all three methods during the second experiment. All samples from both experiments were analyzed for the presence of Round Goby (*Neogobius melanostomus*) DNA, a high-profile invasive fish that was first identified in the Hudson River in 2021.^16^ Samples were analyzed with five quantitative polymerase chain reaction (qPCR) replicates using the *ReesCOI* marker^15^ at the USGS Northern Rocky Mountain Science Center (NOROCK) in Bozeman, MT.

### Case Study #2: Cherry Creek, Bozeman, MT

We installed the autosampler along the bank of Cherry Creek on the Turner Enterprises, Inc.’s Flying D Ranch, 35 km southeast of Bozeman, MT and approximately 25 km upstream from its confluence with the Madison River. Cherry Creek ranges in elevation from approximately 2652 m at its headwaters, to 1717 m at the study site, and to 1350 m at its confluence with the Madison River. The drainage area and gradient at the location of the autosampler were 79 km^2^ and 1.5%, respectively. During the study, stream width was approximately 3 m and discharge was approximately 0.20 m^3^/s. This stream reach is part of a large native fish restoration project^17^ where non-native Rainbow Trout (*Oncorhynchus mykiss*) and Brook Trout (*Salvelinus fontinalis*) were eradicated from all habitats upstream of a 7-m high waterfall (located 15 km downstream of the autosampler) and replaced with native Westslope Cutthroat Trout (*Oncorhynchus clarkii lewisi*).

We ran two experiments which mostly mirrored the Hudson River deployment. In the first, the autosampler was programmed to collect eight samples per day (one every three hours) for seven consecutive days from August 14–21, 2023. In the second experiment, we collected a daily pair of 2-L grab samples concurrently alongside the midday sample collected by the autosampler (as part of the first experiment) for a total of seven triplicate samples. The grab samples were transported on ice to the USGS NOROCK laboratory where they were filtered 1–3 h after collection with a peristaltic pump using (a) 5-μm PES filters, and (b) 1.5-μm glass-fiber filters and extracted immediately. We collected one field control with the autosampler immediately prior to the first field sample. We collected grab sample field controls for the second experiment at the start and end of the autosampler deployment by filtering 250 mL of reverse osmosis water through PES and glass-fiber filters following the same procedures of the field samples. All samples from both experiments were analyzed with five qPCR replicates for the presence of Westslope Cutthroat Trout DNA using the *NADH* marker^18^ at the USGS NOROCK laboratory.

We also deployed barometric pressure transducers (Onset HOBO water-level data logger U20-001-01, Bourne, MA) set to 1-h intervals in the air and water to monitor changes in water-surface elevation (hereafter water level) as a proxy for discharge throughout the experiment.

### Case Study #3: Loggers Creek, Boise, ID

The autosampler was installed along the bank of Loggers Creek in Boise, ID, approximately 2.4 km upstream from its confluence with the Boise River. Loggers Creek is a 5-km long, shallow, low-gradient stream in the Boise River floodplain that meanders through housing developments just east of downtown Boise at an elevation of 830 m. During the study, stream width was approximately 3 m and discharge was approximately 0.67 m^3^/s. The autosampler was positioned approximately 10 m downstream of a translocated bed of 41 Western Pearlshell mussels (*Margaritifera falcata*). The mussels were moved to this location from a downstream reach of Loggers Creek in advance of a bridge construction project 2 weeks prior to autosampler deployment. Western Pearlshell is a native unionid mussel to the Boise River watershed facing population decline while Rainbow Trout are one of the hosts for Western Pearlshell and are ubiquitous in the area.

The autosampler completed two discrete collections, taking eight samples per day (one every three hours) from July 26–28, 2023 and from August 1–3, 2023, for a total of 31 samples. We collected one field blank with the autosampler on August 3 after the last field sample was collected. Additionally, we collected six manual samples and one field control at the intake of the autosampler with an eDNA backpack sampler using the same 5-μm self-preserving PES filters. All samples were analyzed with three qPCR replicates each for the presence of Rainbow Trout and Western Pearlshell DNA using the markers described in Wilcox et al.^18^ and Dysthe et al.,^19^ respectively, at the USGS Pacific Northwest Environmental DNA Laboratory in Boise, ID.

### Case Study #4: Big Piney River, St. Robert, MO

The autosampler was positioned along the bank of the Big Piney River, 6.5 km east of St. Robert, MO. The Big Piney River is in the Ozark Highlands region and is part of the Missouri River watershed, flowing northeast 177 km from its headwaters near Cabool, MO to its confluence with the Gasconade River. The autosampler was located near river kilometer 13 where the river has an approximate elevation of 230 m, width of 40 m, and drainage area of 1878 km^2^. The average annual discharge at the nearest USGS gaging station (USGS 06930060 Big Piney below Fort Leonard Wood, MO), located approximately 19 km upstream of the autosampler location, is 20.5 m^3^/s.^20^

The autosampler was used on a single day (September 26, 2023) as part of a larger eDNA fate and transport experiment that incorporated the autosampler into a network of manual grab sampling stations. In this experiment, a frozen block of Lake Trout (*Salvelinus namaycush*) slurry was placed in the middle of the river 600 m upstream of the autosampler. Lake Trout are not present in the watershed so this slurry release represented a novel DNA source. The slurry was placed at the downstream end of a known bed of Spectaclecase mussels (*Cumberlandia monodonta*), which is a federally endangered species. The slurry was placed in the river at 9:00 am and by 11:00 am the block was mostly melted. The autosampler began sampling at 9:04 am and sampled approximately every 15 min, collecting a total of 16 samples throughout the day. The first sample acted as a de facto field control for Lake Trout as the melting slurry was not expected to reach the autosampler in 4 min; however, no true field controls were taken during this deployment. The first eight samples from the autosampler were set to filter 2 L and the second batch of eight samples was set to filter 4 L of water to explore autosampler filtration capability. The 16 samples from the autosampler were compared with results from nine paired grab samples taken approximately 2 m downstream of the autosampler. Each grab sample was composed of four 50-ml field replicates that were transported on ice to the laboratory where they were refrigerated and then centrifuged in the laboratory within 48 h of collection. All samples were analyzed with four qPCR replicates each for the presence of Lake Trout DNA using the assay from Kronenberger et al.^21^ and Spectaclecase DNA using the alternate COI marker 2 for Spectaclecase from Lor et al.^22^ at the USGS Columbia Environmental Research Center (CERC) in Columbia, MO.

### Data Analysis

Across all four case studies, a sample was considered positive for the target DNA if at least one qPCR replicate amplified prior to the maximum number of cycles. We used a liberal definition of a positive sample because the target species were known to be present at the sites and our aim was to evaluate autosampler efficacy rather than infer organismal presence. DNA concentrations were estimated as copies per liter (copies/L) for all positive samples using standard curves and in reference to elution volume and the original sample volume. Samples in which no qPCR replicates amplified were considered negative for the target species and were assigned a DNA concentration of 0 copies/L following guidance from Ellison et al.^23^ The full suite of data and metadata from each case study, including details on DNA extraction and other laboratory conditions and methods, is available in Sepulveda et al.^24^

For the three case studies in which the autosampler conducted 24-h sampling regimes, all samples were classified as “daylight” or “darkness” for subsequent comparisons using local sunrise and sunset times. Additionally, all samples were classified as having filtered to completion or terminated early due to filter clogging prior to reaching target volume. Any sample that reached or exceeded 1.88 L was considered to have filtered to completion based on the manufacturer’s estimated volume measurement error (up to 5%) and an observed natural break in the volume data collected in the case studies. Additionally, for the Hudson River case study we explored the relation between turbidity and sample volume by pairing each recorded filtration volume with the closest measured turbidity value (within 7 min for all but two pairings). We then used one-inflated β regression with the “zoib” package ver. 1.6^25^ in R version 4.0.5^26^ to relate the proportion of target volume filtered to turbidity, with turbidity effects on both the expected value of the β and one-inflation (Bernoulli) submodels. This distribution accommodated (a) the proportion data for samples where the full target volume was not achieved through the β submodel (which does not allow values of 1) and (b) the samples that achieved (and were capped at) the full target volume through the Bernoulli one inflation submodel.

To benchmark the efficacy of the autosampler against established eDNA methods, we compared DNA concentrations in autosamples and manually collected samples. All results were evaluated as average copies per L to account for variation in water volume filtered. The effects of environment covariates (e.g., discharge, turbidity, temperature) were assumed to be similar within a set of paired samples, since autosamples and manual samples were collected at the same place and approximately same time. These comparisons were done using basic summary statistics for all four case studies and with analysis of variance (ANOVA) of log-transformed DNA concentration for the Hudson River and Cherry Creek case studies which provided sufficient sample size and satisfied statistical assumptions. At the Hudson River site, we compared the autosampler to the backpack sampler, excluding the hand pump from analysis due to an abundance of nondetections which violated the assumption that residuals were normally distributed. At Cherry Creek, we compared the autosampler, manual sampling with PES filters, and manual sampling with glass fiber filters using Tukey’s Honest Significant Difference Method to adjust for multiple comparisons. In both analyses, we controlled for sampling time by included it as a fixed factor.

## Results

### Case Study #1: Hudson River, Albany NY

**Figure 2:**
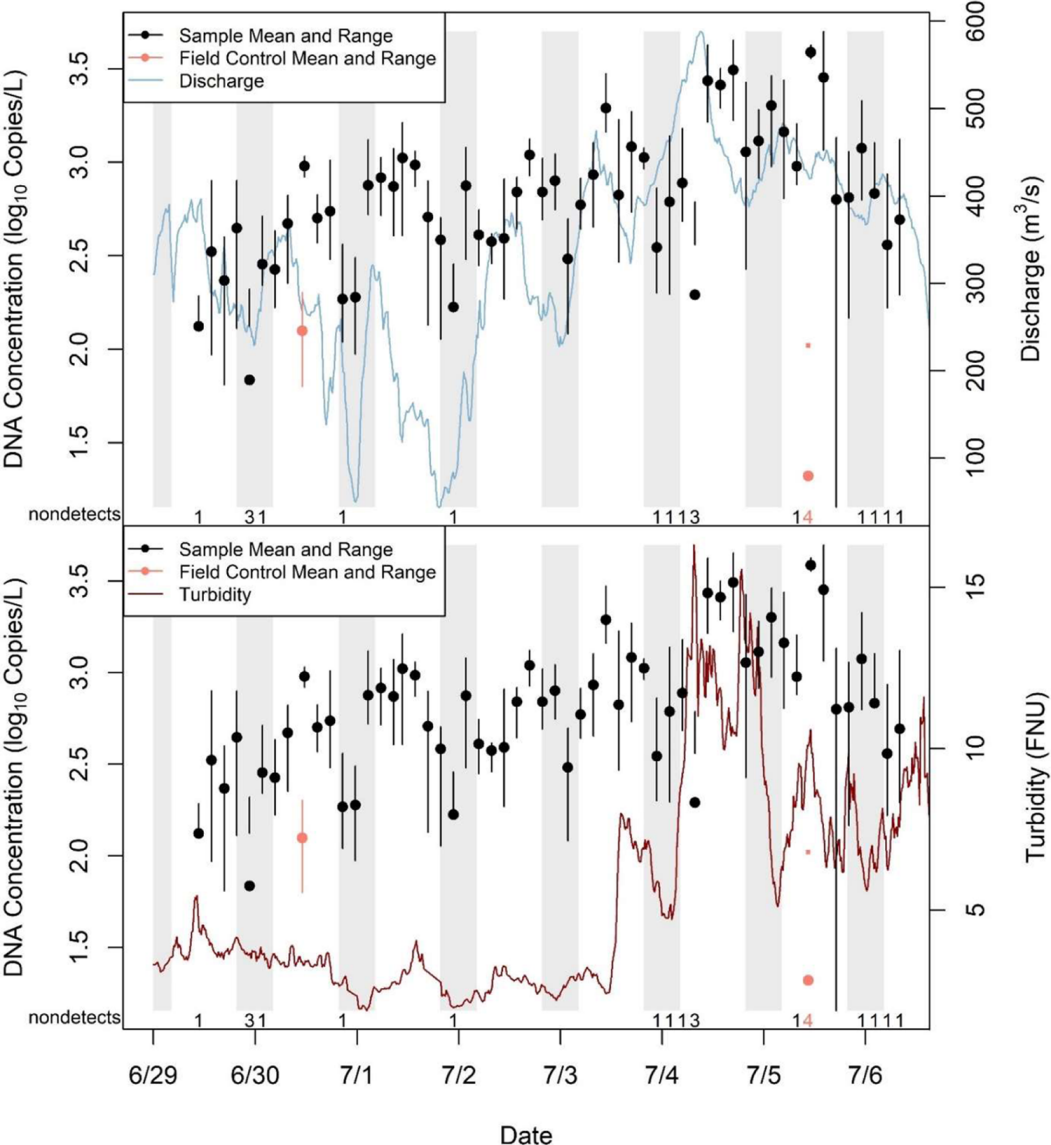
Mean and range of eDNA concentrations (five PCR replicates) from 56 samples (black) and two field controls (pink) collected by the autosampler in the Hudson River case study (June 29–July 6, 2023) plotted against a 1-h running average of continuous (15 min) discharge (top panel) and turbidity data (bottom panel). The number of qPCR replicates for a given sample that did not amplify are listed above the *x*-axes and were assigned a zero for sample mean calculation. Vertical shading denotes periods of darkness.

The autosampler successfully collected all 56 of the intended samples during the seven-day monitoring period. Filtered volumes ranged from 0.6–1.93 L, and 48% of samples were classified as having filtered to completion. Round Goby DNA was detected in 56 of the 56 samples (100%) and the mean DNA concentration ranged from 68 to 3873 copies/L and averaged 888 copies/L (Figure 2). The two field controls taken during this experiment had detectable concentrations of Round Goby DNA. In the June 30 control taken on the second day of the deployment, all five qPCR replicates were positive with a mean concentration of 125 copies/L. In the July 5 control taken on the seventh day of the deployment, one of five qPCR replicates was positive and the sample had a mean concentration of 21 copies/L.

There was no discernible difference in detection rates between samples taken during the daylight (*n* = 35, 100%) and darkness hours (*n* = 21, 100%, Figure 2). The mean DNA concentration and variability between samples were greater in daylight samples (1022 copies/L, standard deviation (SD):926 copies/L) compared to darkness samples (666 copies/L, SD:472 copies/L).

The relation of DNA concentration with discharge and turbidity was complex. A three-day rain event occurred between July 2–4 totaling 41.4 mm of rain as recorded at Albany International Airport.^27^ Discharge and turbidity peaked on July 4 and remained elevated for the rest of the deployment (Figure 2). On the initial rising limb of the hydrograph on July 4, a brief decline was observed in eDNA concentration (including a sample in which three of five qPCR replicates were nondetects) but then eDNA concentration rose abruptly and remained at the highest levels observed in the deployment during the ensuing period of peak discharge and turbidity. The mean eDNA concentration in the 16 samples taken after the peak discharge on July 4 was 1624 copies/L, compared to 594 copies/L in the 40 preceding samples. Additionally, discharge exhibited erratic daily peaks, likely due in part to operations of hydroelectric facilities in the system. The concentration of eDNA was generally high in samples taken immediately following the daily peaks in discharge.

**Figure 3:**
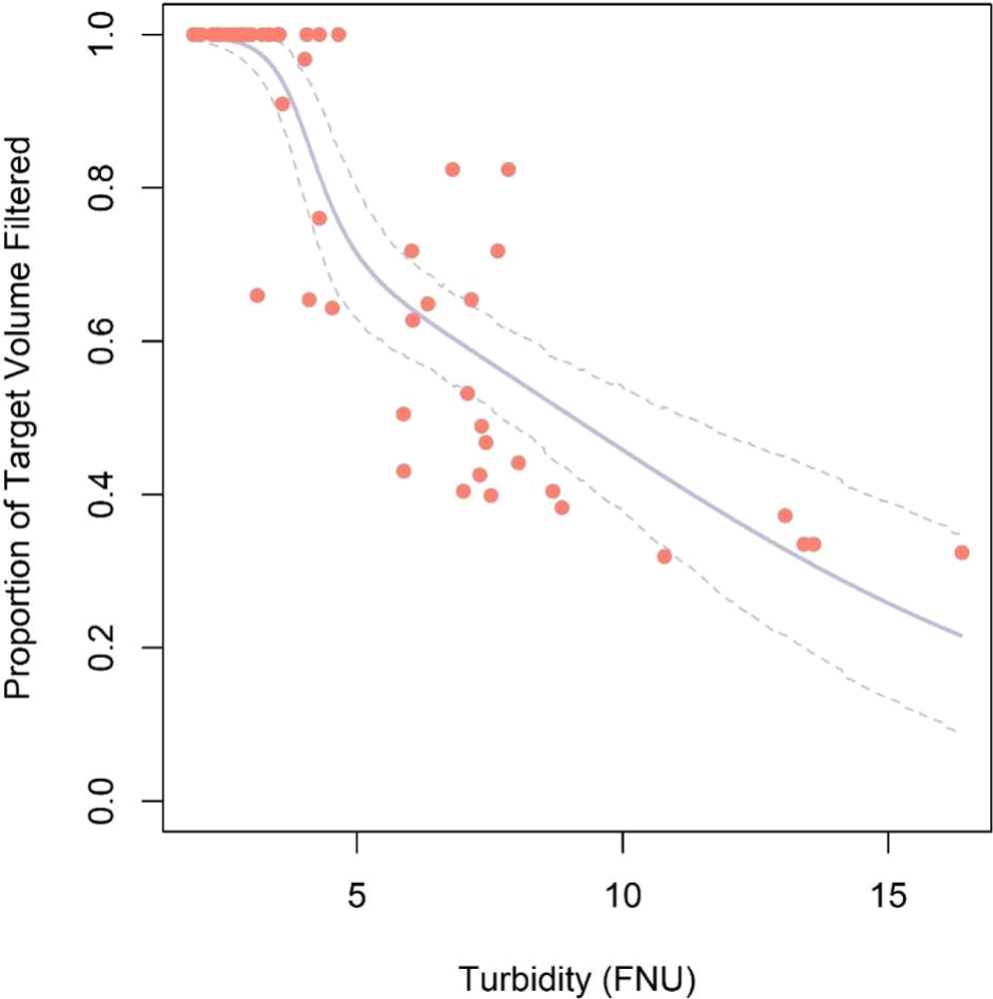
Estimated relation (with 95% confidence interval) between the proportion of target volume filtered and turbidity from a one-inflated β regression for 56 samples collected by the autosampler in the Hudson River case study.

The volume of water the autosampler filtered for a sample was negatively related to turbidity. Twenty-seven of the 33 samples (82%) taken when turbidity was <5 formazin nephelometric units (FNU) were classified as having filtered to completion, while none of the 23 samples taken when turbidity was >5 FNU filtered to completion (Figure 3). The slopes from the one-inflated β regression relating turbidity to (a) the proportion of sample volume filtered and (b) the probability of filtering to completion were estimated to be −0.18 (95% confidence interval (CI): −0.27 to −0.10) and −2.71 (95% CI: −4.71 to −1.35), respectively. The posterior probability of a negative effect of turbidity on the response of both submodels was 1.00 and the effect size was large—the proportion of target sample volume filtered was estimated to decline from 1.00 to around 0.20 across the range of observed turbidity (Figure 3).

**Figure 4:**
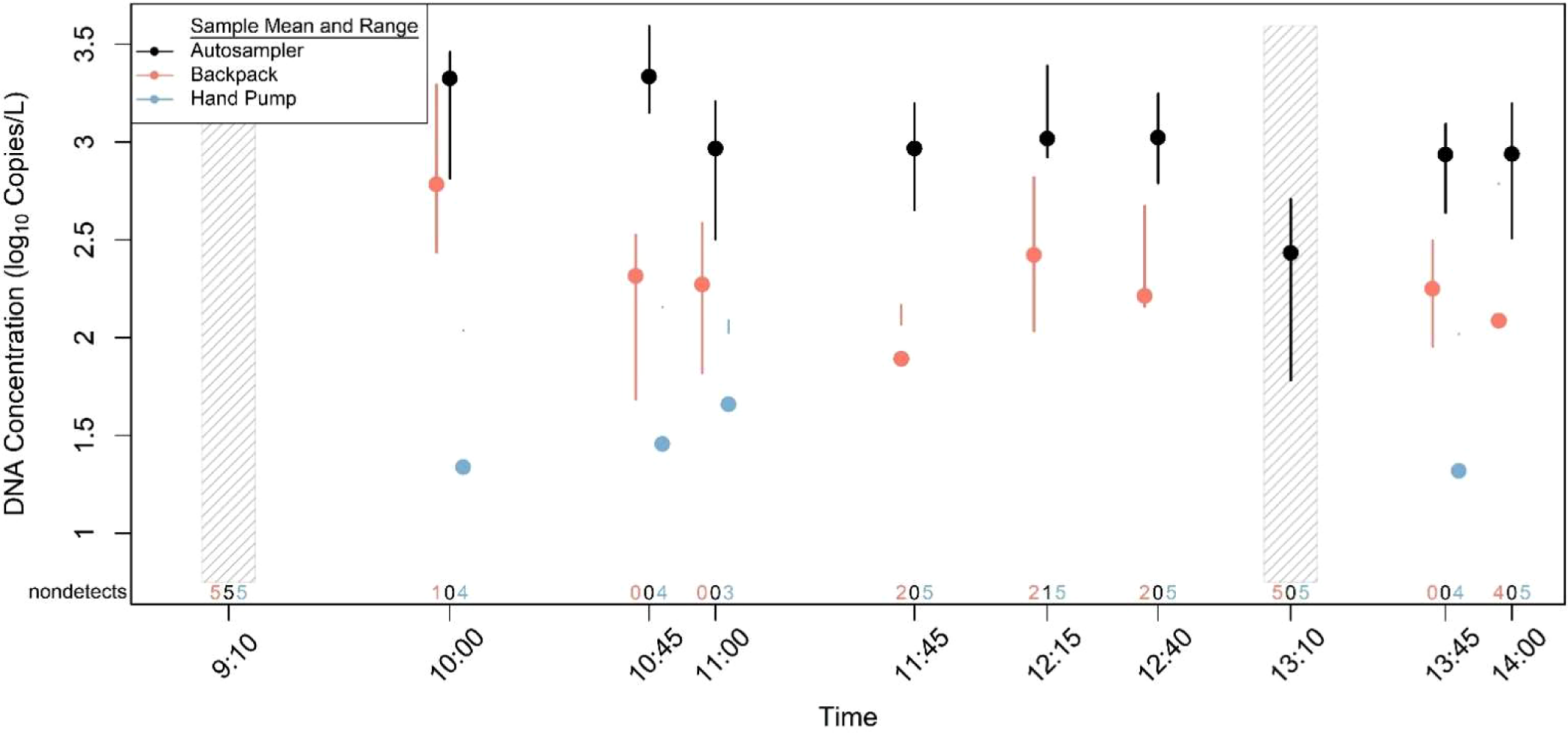
Mean and range of eDNA concentrations (five PCR replicates) from eight samples and two field controls collected in triplicate using the autosampler, backpack sampler, and manual hand pump in the Hudson River case study. The number of qPCR replicates for a given sample that did not amplify are listed above the *x*-axis and were assigned a zero for sample mean calculation. Dashed regions denote paired field controls.

Large differences were observed in both the detection rate and DNA concentration between the autosampler, backpack sampler, and manual handpump during the method triplicates experiment. The autosampler and backpack each produced detections for eight out of eight samples while the handpump produced detections in four of the eight samples (Figure 4). The number of positive qPCR replicates per sample (out of a possible 5) averaged 4.9 for the autosampler, 3.6 for the backpack, and 0.6 for the handpump. The mean eDNA concentration varied significantly by method (*F*_1,7_ = 133.61, *p* < 0.0001) and time (*F*_7,7_ = 4.16, *p* = 0.0399), with the autosampler obtaining higher DNA concentration than the backpack sampler. The mean concentration of DNA was 1245 copies/L (SD:555) in filters from the autosampler, 226 copies/L (SD:164) in filters from the backpack, and 15 copies/L (SD:17) in filters from the handpump. The two field controls taken with the backpack and handpump produced nondetections, while one of the two field controls taken by the autosampler produced a nondetection and the other had all five qPCR replicates positive with a mean concentration of 271 copies/L.

### Case Study #2: Cherry Creek, Bozeman, MT

**Figure 5:**
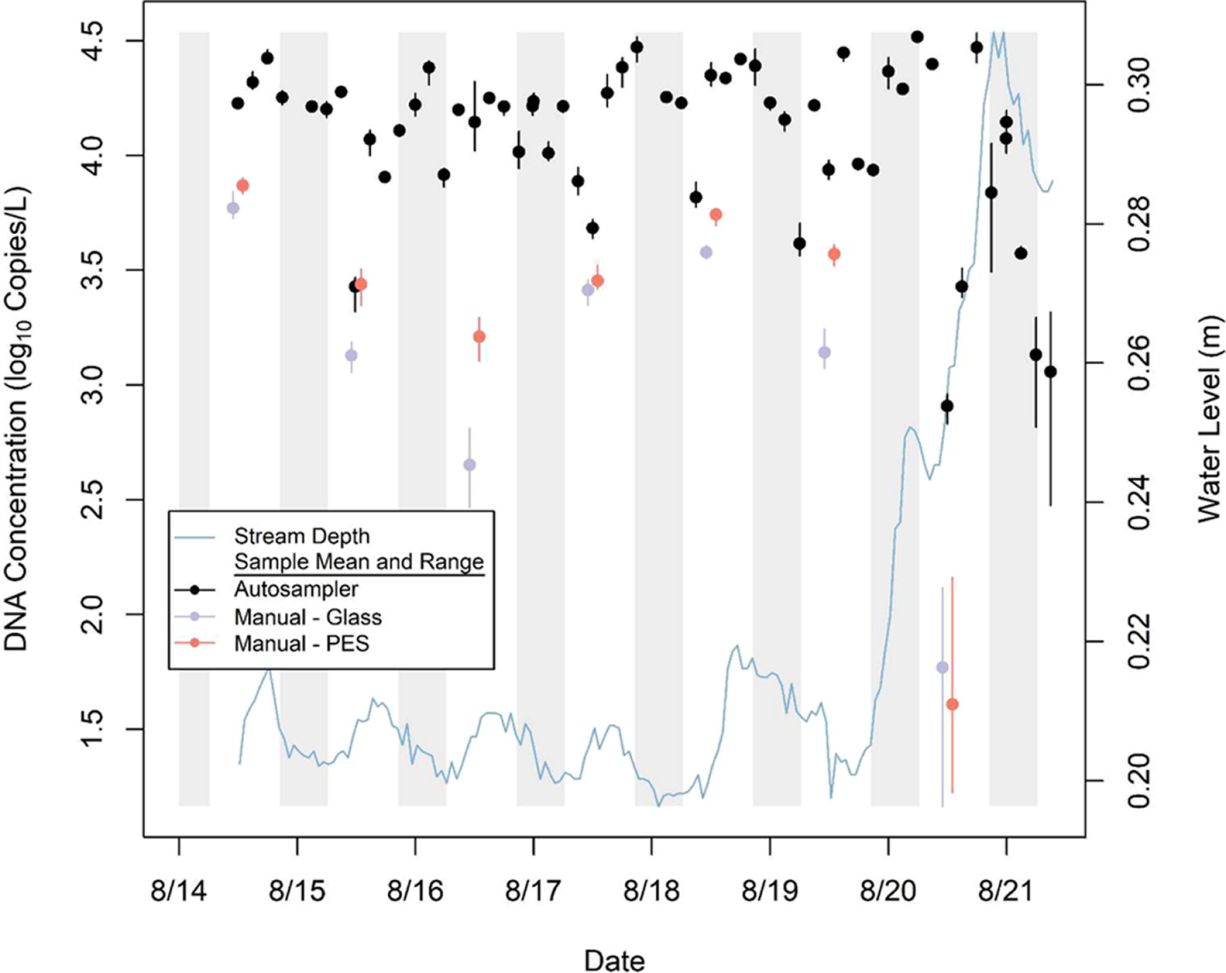
Mean and range of eDNA concentrations (five PCR replicates) from 56 samples collected by the autosampler and seven pairs of manual grab samples (Glass: glass-fiber filter, PES: polyethersulfone) in the Cherry Creek case study (August 14–21, 2023) plotted against 1-h water level. Vertical shading denotes periods of darkness.

The autosampler successfully collected all 56 of the intended samples during the seven-day monitoring period. Filtered volumes ranged from 0.29–2.03L, and 79% of samples were classified as having filtered to completion. Westslope Cutthroat Trout DNA was detected in 56 of the 56 samples (100%) and the mean DNA concentration ranged from 809 to 32,809 copies/L and averaged 15,205 copies/L (Figure 5). The field control taken on August 14 immediately prior to sample collection produced a nondetection.

Detection rates of samples taken during daylight (*n* = 28, 100%) and darkness hours (*n* = 28, 100%) were identical (Figure 5). There were also no practical differences between eDNA concentrations of these samples. We detected an average of 15,107 copies/L (SD: 8732 copies/L) during daylight and 15,305 copies/L (SD: 7381) during night.

A decline in eDNA concentration was associated with a rain event near the end of the deployment (Figure 5). Between August 19–21, 22.4 mm of rainfall was recorded at the nearest publicly available rain gauge^28^ and the measured water level at the autosampler increased by approximately 10 cm. Similar to the Hudson River deployment, eDNA concentration declined during the rising limb of the hydrograph with one sample in that period producing the lowest concentration observed in the entire deployment. Once the hydrograph peaked, eDNA concentrations were generally low and also highly variable between and within (PCR replicates) samples. The three lowest eDNA concentrations occurred in samples after the hydrograph began rising and the mean concentration of the last nine samples collected (taken during the highest part of the hydrograph) was 9456 copies/L (SD: 10,955) compared to 16,307 copies/L (SD: 6940) for the prior 47 samples.

Target eDNA was detected in all samples in the method triplicates experiment regardless of the sampling method; however, we observed large differences in eDNA concentration among sampling methods (Figure 5). Mean eDNA concentration differed significantly by method (*F*_2,12_ = 13.46, *p* = 0.0009) and time (*F*_6,12_ = 14.81, *p* < 0.0001), with the autosampler producing higher concentrations than the manual method with PES filters (*p* = 0.0088) and glass filters (*p* = 0.0008). Mean eDNA concentration across the seven samples from the autosampler was 10,020 copies/L (SD: 7981 copies/L), while the mean eDNA concentrations across the seven grab samples filtered with either 5-μm PES or 1.5-um glass-fiber filters was 3409 (SD: 2434) and 2212 (SD: 2053) copies/L, respectively. We did not detect a significant difference between the filter types for manual sampling (*p* = 0. 3787).

### Case Study #3: Loggers Creek, Boise, ID

**Figure 6:**
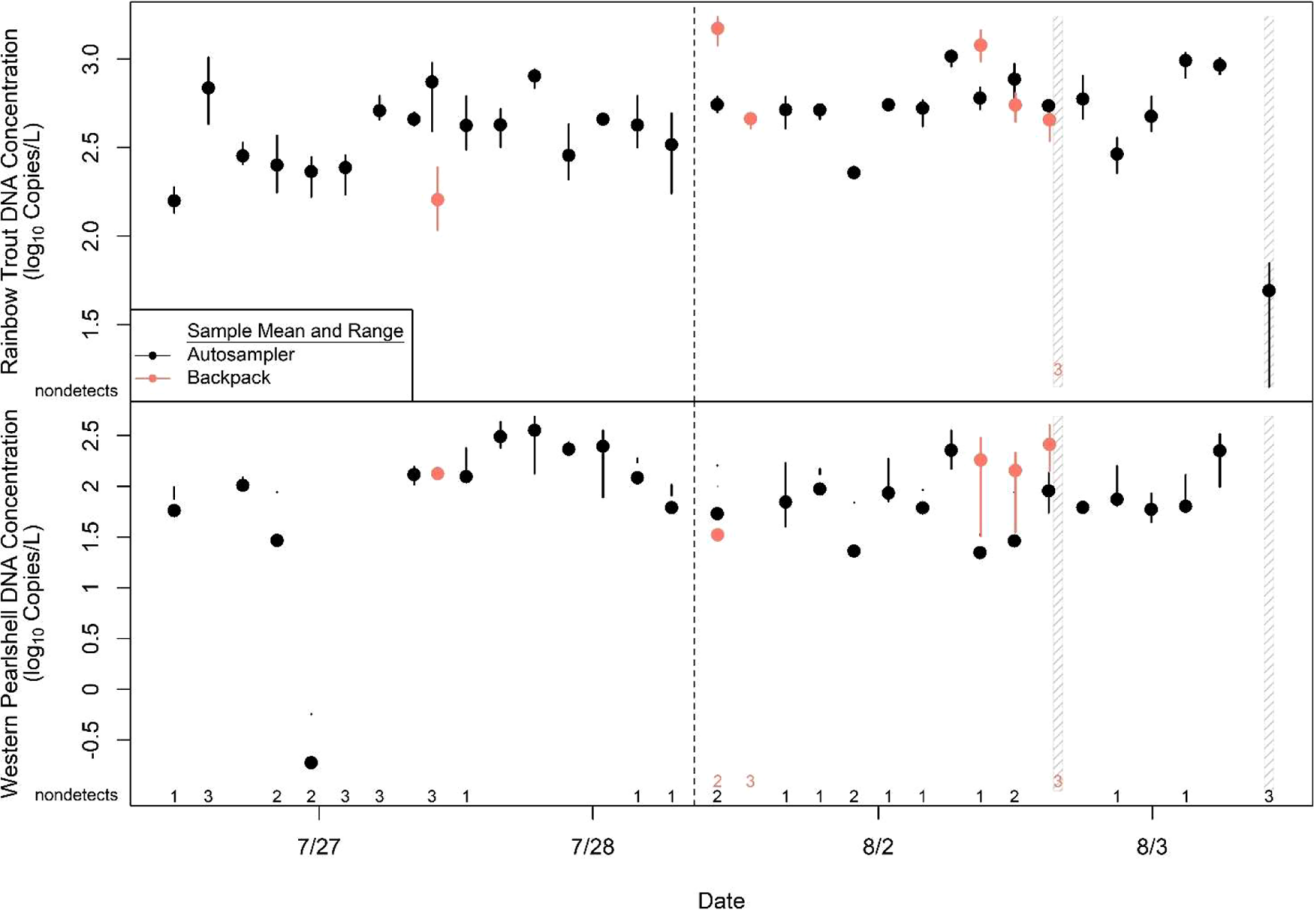
Mean and range of eDNA concentrations (3 PCR replicates) from 31 samples and 1 field control collected by the autosampler, and 6 paired samples and 1 field control collected with the backpack sampler in the Loggers Creek case study. The number of qPCR replicates for a given sample that did not amplify are listed above the *x*-axes and were assigned a zero for sample mean calculation. Vertical dashed line indicates a break in the time series. Dashed regions denote paired field controls.

The autosampler successfully collected all 16 of the intended samples across the first monitoring period and 15 of the 16 intended samples during the second monitoring period. The failed sample occurred on August 1 in which water backed up inside the filter housing. That sample was excluded from subsequent analyses because it was unclear what volume had passed through the filter and if it was preserved adequately. Filtered volumes ranged from 1.58–2.02 L, and 87% of samples were classified as having filtered to completion. Rainbow Trout DNA was detected in 31 of the 31 samples (100%) and the mean DNA concentration ranged from 158 to 1039 copies/L and averaged 511 copies/L (Figure 6). Western Pearlshell DNA was detected in 27 of the 31 samples (87%) and the mean DNA concentration ranged from 0 to 356 copies/L and averaged 97 copies/L (Figure 6). The field control taken on August 3 following the last field sample did not amplify for Western Pearlshell but all three qPCR replicates were positive for Rainbow Trout DNA with a mean concentration of 49 copies/L.

Detection rates for each species were nearly identical in samples taken during daylight (*n* = 18, 100% for Rainbow Trout, 89% for Western Pearlshell) and darkness hours (*n* = 13, 100% for Rainbow Trout, 85% for Western Pearlshell). Concentrations of eDNA were also similar between daylight and darkness samples for each species although minimally higher concentrations and variability between samples occurred in darkness samples (Figure 6). For Rainbow Trout, the mean DNA concentration was 497 copies/L (SD: 187 copies/L) in daylight samples compared to 529 copies/L (SD: 283 copies/L) in darkness samples. For Western Pearlshell, the mean DNA concentration was 93 copies/L (SD: 95 copies/L) in daylight samples compared to 104 copies/L (SD: 97 copies/L) in darkness samples.

In the method duplicates experiment, the six manual samples collected with the backpack sampler had a detection rate of 100% for Rainbow Trout and 83% for Western Pearlshell DNA (Figure 6). The mean eDNA concentration of the manual samples was 719 copies/L (SD: 510 copies/L) for Rainbow Trout and 125 copies/L (SD: 95 copies/L) for Western Pearlshell DNA, compared to 629 copies/L (SD: 102 copies/L) and 42 copies/L (SD: 32 copies/L), respectively, in the six most-closely paired samples collected by the autosampler. The field control taken with the backpack sampler was negative for both targets.

### Case Study #4: Big Piney River, St. Robert, MO

The autosampler successfully collected all 16 of the intended samples during the four-hour monitoring period. Filtered volumes of the first eight samples (2-L target) ranged from 1.94–2.03 L and 100% of samples were classified as having filtered to completion, while filtered volumes of the next eight samples (4-L target) ranged from 3.95–3.99 L.

**Figure 7:**
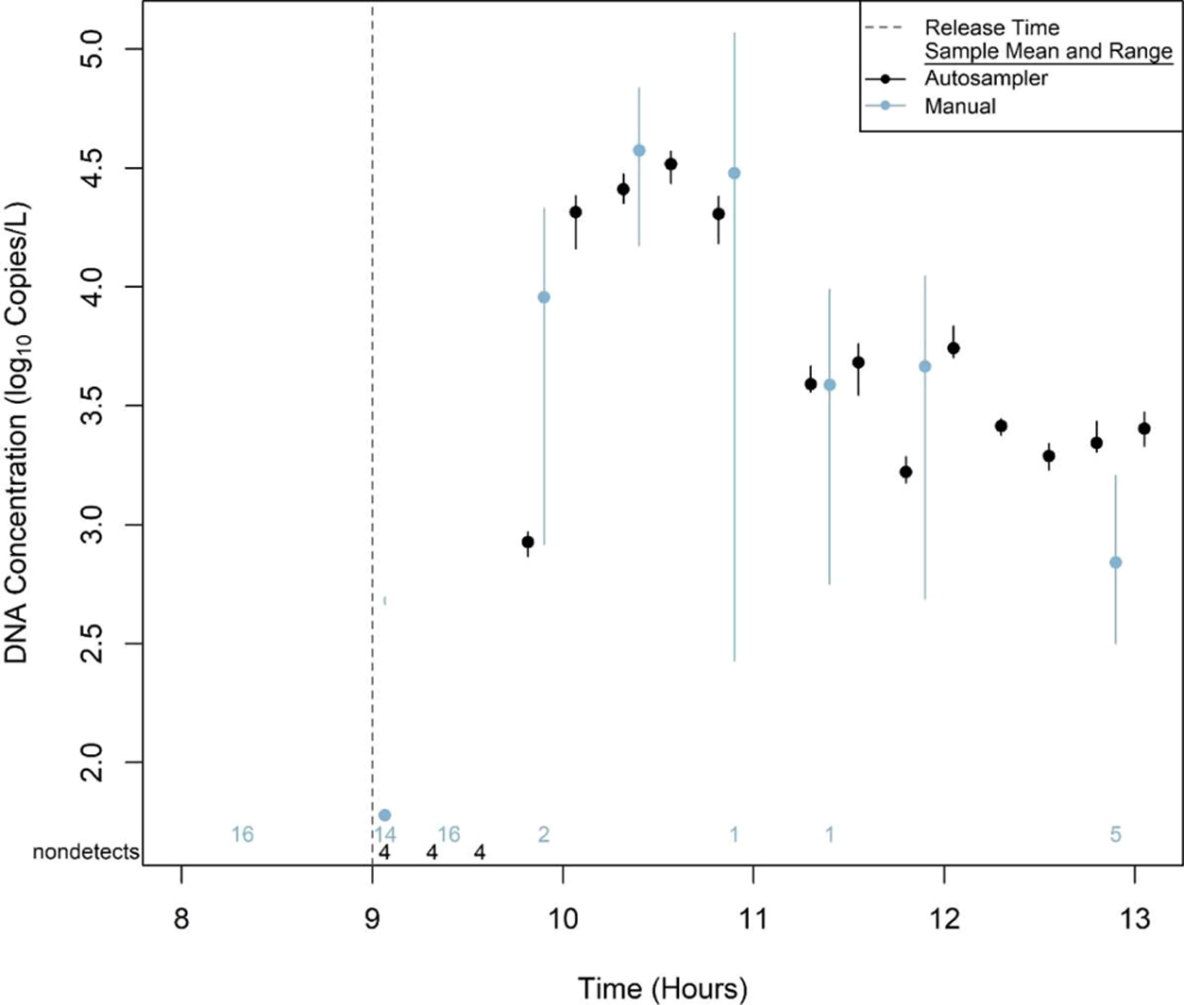
Mean and range of eDNA concentrations (four PCR replicates) from 16 samples collected by the autosampler and nine paired manual centrifuge samples (collected in quadruplicate) during the Lake Trout slurry addition in the Big Piney River case study. Vertical dashed line indicates the time point at which the frozen slurry was added. The number of qPCR replicates for a given sample that did not amplify are listed above the *x*-axis and were assigned a zero for sample mean calculation.

Lake Trout DNA was detected in 13 of the 16 samples collected by the autosampler. The three samples that produced nondetections were the first three of the experiment, occurring within 34 min of the slurry release. The mean DNA concentration ranged from 0 to 32,802 copies/L. Similarly, Lake Trout DNA was detected in 7 of the 9 grab samples, and the two nondetections occurred within the first 30 min of the release. The mean DNA concentration of the grab samples ranged from 0 to 37,363 copies/L. Due to the pulsed release of Lake Trout eDNA in the system, we expected and did observe an initial absence, rapid increase, and subsequent decrease in eDNA concentration over the course of the experiment. Given this instability, comparisons between the autonomous and grab samples are best made with pairs of samples collected at approximately the same time, rather than groups of samples over time (Figure 7). These pairwise comparisons generally indicated similar detection patterns and DNA concentrations between the two methods.

Spectaclecase DNA was detected in 7 of the 16 samples collected by the autosampler and DNA concentrations were consistently low, ranging from 0 to 84 copies/L and averaging 14 copies/L. In the paired grab samples, only one of the nine samples detected Spectaclecase DNA with a mean value of 55 copies/L. The one positive grab sample resulted from a single positive PCR replicate in one of the four 50 mL field replicates.

## Discussion

We successfully deployed an eDNA autosampler in four lotic freshwater ecosystems across the contiguous United States. These trials involved environments ranging from a headwater stream at over 1700 m of elevation to one of the largest rivers in the eastern United States at an elevation close to sea level. In all four case studies, the autosampler followed a programmed sampling regime and collected samples that amplified for the target DNA sequences. In general, it took the autosampler 10–15 min to collect an individual 2 L sample, with approximately 6 min required for the 5-L flush and the remaining time to complete filtration. During the multiday deployments, at least two technicians were needed to deploy and retrieve the autosampler and a single technician was needed to maintain the autosampler during routine visits. For maintenance, a technician typically spent less than an hour on site to retrieve and label the eight spent filters, reload the autosampler with new filters, program the next sampling regime, and periodically check the water intake for biofouling. These tasks were completed by technicians with no specialized background and minimal training, indicating that this technology can better democratize high quality, biological data collection by making standardized eDNA sample collection accessible to more groups.^29^

The high-frequency autosampling enabled a number of analyses and comparisons that would otherwise be difficult with manually collected eDNA samples. First, three case studies addressed the question of “What are we missing at night?” by collecting routine samples around the clock. Little is known about diurnal trends in eDNA concentrations because most eDNA monitoring occurs during daylight hours.^30^ The DNA concentration was nearly identical between daylight and darkness samples in the Cherry Creek and Loggers Creek case studies, while the Hudson River case study found a 42% greater DNA concentration in daylight samples. It is not clear if the hydraulic dynamics in this larger system create a different diel eDNA signature from the other case studies, if this reflects differences in target organism activity or physiology, or if this result is simply random noise from a short-term deployment. However, the measured environmental covariates at this streamgage are helpful for interpreting the observed temporal dynamics in eDNA concentration.

Environmental covariates can influence eDNA distribution and concentration but the magnitude and direction of these forces are difficult to discern without paired, high-resolution eDNA and environmental data. Our time series demonstrate how high frequency sampling made possible by autosamplers increases the potential to identify environmental variables like discharge, precipitation, and turbidity that influence eDNA dynamics. Co-locating autonomous eDNA samplers in streamgages is one effective approach for obtaining high resolution environmental data.^8,9^ In the Hudson River streamgage deployment, eDNA concentration appeared positively related to discharge and/or turbidity at the interday scale and potentially also with discharge at the intraday scale. This data set also clearly demonstrated the negative effect of turbidity on filtering capacity (Figure 3). In contrast, eDNA concentration appeared negatively related to elevated water level from a precipitation event in the Cherry Creek deployment (Figure 5). Other studies have observed highest eDNA concentrations during moderate-discharge periods^10^ and depressed eDNA concentrations during high-discharge periods.^31,32^ The complexity and inconsistency of this relation observed in our case studies likely reflects differences in stream size and associated hydraulic dynamics–the Cherry Creek site had a drainage area and approximate width of 79 km^2^ and 3 m, respectively, compared to 20,953 km^2^ and 280 m at the Hudson River site. We hypothesize that abiotic processes, such as resuspension of deposited or sediment-bound eDNA,^33,34^ may have contributed to the positive relation between DNA concentration and discharge and turbidity in the Hudson River deployment. However, the role of biotic processes such as differences in species-specific behavorial responses to changes in flows cannot be ruled out. Ultimately, longer time series are needed to disentangle the effect of these covariates on eDNA concentration.

In general, more is known about factors contributing to spatial variation in eDNA concentrations than temporal variation,^35^ and we were hopeful that the autosampler could fill some of this information gap. The high variability in eDNA concentrations observed in our case studies, however, suggests that longer time series alone may not be adequate to discern signal from noise without increased focus on temporal replication. Variation between samples was sometimes observed in the 1000s of copies/L, and standard deviations approached mean eDNA concentrations in some case studies. This degree of variability is problematic, especially during short (i.e., 1 week) deployments during which the population dynamics of target species are likely static. The polydisperse nature of eDNA molecules, measurement error, and PCR stochasticity make high variability in eDNA concentrations a common attribute of many eDNA studies.^32,36^ This variability can result in the noise associated with sampling variation being greater than the signal of interest (e.g., response of eDNA to changes in species behavior), and also makes it challenging to identify influential environmental covariates. Temporal replication is needed to filter out the noise, but such replication is difficult and costly to achieve with autosamplers. The autosampler we used cannot collect true replicates (i.e., multiple samples collected at the same point in space and time); rather it is limited to sequential samples separated by approximately 15 min. Sequential samples may provide some insight about intersample variability, but at the expense of rapidly expending the 8-sample capacity of the autosampler and increasing the frequency of technician visits. Alternative strategies for acquiring replicates might include colocating multiple autosamplers or leveraging routine technician visits to collect sequential samples with the autosampler or multiple manual samples. Future iterations of autonomous eDNA samplers could mitigate this problem by considering design specifications that enable replicate sampling, minimize the time interval between sequential samples, or increase the sample capacity.

Our benchmark assessments found that autosampling resulted in similar detection patterns as manual sampling, but comparisons of eDNA concentration were variable among case studies. For the Hudson River and Cherry Creek case studies, we observed significantly and consistently higher eDNA concentration in the autosamples. The mean DNA concentration obtained from the paired autosamples was 5.5- and 83-times greater than backpack and handpump samples, respectively, in the Hudson River trial, and over 2.9- and 4.5- times greater than the manual sample types in the Cherry Creek trial. In contrast, the Loggers Creek and Big Piney River trials found similar DNA concentrations between auto- and manual samples. The stark differences in DNA concentration between autonomous and manual samples in the Hudson River and Cherry Creek case studies cannot be explained by filter material or filter preservation, as both studies used 5-μm PES filter material for autosampling and manual sampling, and the Hudson River case study even used the backpack sampler where sample desiccation began immediately after filtration similar to the autosampler. Our sample sizes were not large so we cannot eliminate the potential that this was a random outcome, but we consider that an unlikely explanation since this outcome occurred consistently throughout two independent case studies. This finding was also comparable to results of a marine eDNA metabarcoding evaluation of a different autosampling platform, which found that autonomous methods had a higher mean number of metabarcoding reads per sample and yielded more sequence data than manually filtered samples, though the mechanisms driving these differences were not fully determined.^37^ Similarly, our case studies were not designed to directly identify causes for differences between collection methods. Focused experiments and additional field trials are needed to evaluate why and in what situations autosamplers provide different results than manual sampling.

One potential limitation of autonomous eDNA sample collection identified in this effort is carryover contamination between samples. The single autosampler field control taken prior to sample collection was negative for target eDNA, but four of the five autosampler field controls taken during deployments were positive for target eDNA despite a preceding 5-L flush of the system with deionized water. This finding likely indicates the presence of residual DNA within the water intake screen, external water lines, or internal components. The DNA concentations obtained in field controls were consistently low, often representing a decrease of an order of magnitude or more from concentrations observed in the preceding and proceeding field samples and did not progressively accumulate with successive field samples. These results mirror those observed in marine environments using a different autosampler, in which postdeployment field controls found residual eDNA^38,39^ at concentrations orders of magnitude below that of routine field samples. The results of our field controls suggest that consecutively collected samples may not be completely independent from one another, especially when separated only by hours as in this study. Nonindependence may be a minor concern for applications where detection of a rare or presumed-absent target is the sampling objective, but could be a limitation if the objectives are eDNA quantification or trend analysis. Multiple disciplines, including eDNA science, have developed approaches to account for background “noise” by using method blanks to determine the threshold (i.e., Limit of Blank) above which a result is likely to be derived from the sample rather than contamination or noise.^40^ An alternative to correcting for nonindependence is to prevent it all together by exploring the use of decontaminating approaches, low binding plastics, or designing autosamplers so that they are “filter-forward” and do not share common intake lines (e.g., Hendricks et al.^41^). These results also highlight the importance of using negative controls to identify and address issues that may occur throughout the course of eDNA workflows.^37^ The ideal number and timing of negative controls with autosampling will depend on study objectives; detection of eDNA from a novel organism may only require negative controls at the start of the sampling mission whereas trend analysis may require negative controls periodically during the sampling mission. While positive controls were not used in this study, they should also be considered for long-duration deployments to assess sample stability.

Autosamplers present a novel solution to the challenges of collecting high-resolution, standardized eDNA samples; however, this solution creates new challenges that must be addressed to make autosamplers operational for mainstream biomonitoring applications. The start-up costs of acquiring autosamplers are significantly greater than that of manual eDNA sampling equipment so scalable implementation of autosampler networks is difficult. Moreover, the human labor-costsavings associated with autosamplers that have limited sampling capacity (e.g., eight samples) will accrue slowly since these type of autosamplers will have to be visited more frequently than autosamplers with larger sampling capacities.^8,10^ Autosamplers also enable a higher volume of field samples than was previously feasible, thereby potentially increasing laboratory processing costs (e.g., consumables and human labor) and transferring the workflow bottleneck from the field to the laboratory. Statistically informed study designs, robust quality control and assurance procedures, higher throughput analysis solutions, and even *in situ* analysis capabilities are needed to keep pace with autosampler advancements. Though we found that this autosampler is at a technology readiness level 7 (prototype demonstration in an operational environment)^29^ or greater, further assessments involving longer deployments, different habitats (e.g., lentic, estuary), and sample replication are needed. In conclusion, eDNA autosamplers have the potential to significantly improve biomonitoring at spatial and temporal scales that are relevant to natural resource management and that were previously impractical to obtain.

## References

[ref1] Kelly R. P.; Lodge D. M.; Lee K. N.; Theroux S.; Sepulveda A. J.; Scholin C. A.; Craine J. M.; Andruszkiewicz Allan E.; Nichols K. M.; Parsons K. M.; et al. Toward a national eDNA strategy for the United States. Environ. DNA 2024, 6 (1), e432 10.1002/edn3.432.

[ref2] Trujillo-González A.; Thuo D. N.; Divi U.; Sparks K.; Wallenius T.; Gleeson D. Detection of Khapra beetle environmental DNA using portable technologies in Australian Biosecurity. Front. Insect Sci. 2022, 2, 795379 10.3389/finsc.2022.795379. 38468794 PMC10926498

[ref3] Larson E. R.; Graham B. M.; Achury R.; Coon J. J.; Daniels M. K.; Gambrell D. K.; Jonasen K. L.; King G. D.; LaRacuente N.; Perrin-Stowe T. I.; et al. From eDNA to citizen science: emerging tools for the early detection of invasive species. Front. Ecol. Environ. 2020, 18 (4), 194–202. 10.1002/fee.2162.

[ref4] Duarte S.; Simões L.; Costa F. O. Current status and topical issues on the use of eDNA-based targeted detection of rare animal species. Sci. Total Environ. 2023, 904, 166675 10.1016/j.scitotenv.2023.166675. 37647964

[ref5] Loeza-Quintana T.; Abbott C. L.; Heath D. D.; Bernatchez L.; Hanner R. H. Pathway to Increase Standards and Competency of eDNA Surveys (PISCeS)—Advancing collaboration and standardization efforts in the field of eDNA. Environ. DNA 2020, 2 (3), 255–260. 10.1002/edn3.112.

[ref6] Darling J. A.; Jerde C. L.; Sepulveda A. J. What do you mean by false positive?. Environ. DNA 2021, 3 (5), 879–883. 10.1002/edn3.194. PMC8941663 35330629

[ref7] Scholin C. A.; Birch J.; Jensen S.; Marin R. III; Massion E.; Pargett D.; Preston C.; Roman B.; Ussler W. III The quest to develop ecogenomic sensors: a 25-year history of the Environmental Sample Processor (ESP) as a case study. Oceanography 2017, 30 (4), 100–113. 10.5670/oceanog.2017.427.

[ref8] Sepulveda A. J.; Birch J. M.; Barnhart E. P.; Merkes C. M.; Yamahara K. M.; Marin R. III; Kinsey S. M.; Wright P. R.; Schmidt C. Robotic environmental DNA bio-surveillance of freshwater health. Sci. Rep. 2020, 10 (1), 14389 10.1038/s41598-020-71304-3. 32873867 PMC7462992

[ref9] Sepulveda A. J.; Hoegh A.; Gage J. A.; Caldwell Eldridge S. L.; Birch J. M.; Stratton C.; Hutchins P. R.; Barnhart E. P. Integrating environmental DNA results with diverse data sets to improve biosurveillance of river health. Front. Ecol. Evol. 2021, 9, 620715 10.3389/fevo.2021.620715.

[ref10] Searcy R. T.; Boehm A. B.; Weinstock C.; Preston C. M.; Jensen S.; Roman B.; Birch J. M.; Scholin C. A.; Van Houtan K. S.; Kiernan J. D.; Yamahara K. M. High-frequency and long-term observations of eDNA from imperiled salmonids in a coastal stream: Temporal dynamics, relationships with environmental factors, and comparisons with conventional observations. Environ. DNA 2022, 4 (4), 776–789. 10.1002/edn3.293.

[ref11] Jones D. N.; Clements K. R.; Sepulveda A. J. A workshop to advance invasive species early detection capacity of The Rapid Environ. DNA Assessment and Deployment Initiative & Network (READI-Net). Manage. Biol. Invasions 2024, 15 (1), 159–167. 10.3391/mbi.2024.15.1.10.

[ref12] Thomas A. C.; Nguyen P. L.; Howard J.; Goldberg C. S. A self-preserving, partially biodegradable eDNA filter. Methods Ecol. Evol. 2019, 10 (8), 1136–1141. 10.1111/2041-210X.13212.

[ref13] National Water Information System data available on the World Wide Web (USGS Water Data for the Nation). https://waterdata.usgs.gov/nwis/inventory?site_no=01358000 (accessed January 31, 2024).

[ref14] Levesque V. A.; Oberg K. A. Computing discharge using the index velocity method; U.S. Geological Survey Techniques and Methods 3–A23, 2012. https://pubs.usgs.gov/tm/3a23/.

[ref15] George S. D.; Baldigo B. P.; Rees C. B.; Bartron M. L.; Winterhalter D. Eastward Expansion of Round Goby in New York: Assessment of Detection Methods and Current Range. Trans. Am. Fish. Soc. 2021, 150 (2), 258–273. 10.1002/tafs.10290.

[ref16] Pendleton R.; Berdan R.; George S.; Kenney G.; Sethi S. A. Round Goby captured in a North American estuary: Status and implications in the Hudson River. J. Fish Wildlife Manage. 2022, 13 (2), 524–533. 10.3996/JFWM-22-012.

[ref17] Clancey P. T.; Shepard B. B.; Kruse C. G.; Barndt S. A.; Nelson L.; Roberts B. C.; Turner R. B. Collaboration, commitment, and adaptive learning enable eradication of nonnative trout and establishment of native Westslope cutthroat trout into one-hundred kilometers of Cherry Creek, a tributary to the Madison River, Montana. In Multispecies and Watershed Approaches to Freshwater Fish Conservation, Dauwalter D. C.; Birdsong T. W.; Garrett G. P., Eds.; Vol. Symposium 91; American Fisheries Society, 2019; pp 589–647.

[ref18] Wilcox T. M.; Carim K. J.; McKelvey K. S.; Young M. K.; Schwartz M. K. The dual challenges of generality and specificity when developing environmental DNA markers for species and subspecies of *Oncorhynchus*. PLoS One 2015, 10 (11), e0142008 10.1371/journal.pone.0142008. 26536367 PMC4633235

[ref19] Dysthe J. C.; Rodgers T.; Franklin T. W.; Carim K. J.; Young M. K.; McKelvey K. S.; Mock K. E.; Schwartz M. K. Repurposing environmental DNA samples—detecting the western pearlshell (*Margaritifera falcata*) as a proof of concept. Ecol. Evol. 2018, 8 (5), 2659–2670. 10.1002/ece3.3898. 29531684 PMC5838043

[ref20] National Water Information System data available on the World Wide Web (USGS Water Data for the Nation). https://waterdata.usgs.gov/nwis/inventory?site_no=06930060 (accessed July 31, 2024).

[ref21] Kronenberger J. A.; Wilcox T.; Young M.; Mason D.; Franklin T.; Schwartz M. Large-scale validation of 46 invasive species assays using an enhanced in silico framework. Environ. DNA 2024, 6 (2), e548 10.1002/edn3.548.

[ref22] Lor Y.; Schreier T. M.; Waller D. L.; Merkes C. M. Using environmental DNA (eDNA) to detect the endangered Spectaclecase Mussel (*Margaritifera monodonta*). Freshwater Science 2020, 39 (4), 837–847. 10.1086/711673.

[ref23] Ellison S. L.; English C. A.; Burns M. J.; Keer J. T. Routes to improving the reliability of low level DNA analysis using real-time PCR. BMC Biotechnol. 2006, 6, 33 10.1186/1472-6750-6-33. 16824215 PMC1559608

[ref24] Sepulveda A. J.; George S. D.; Hutchins P. R.; Pilliod D. S.; Klymus K. E. Environmental DNA sampling results used to evaluate and benchmark autosamplers in Idaho, Missouri, Montana and New York rivers 2023; U.S. Geological Survey data release, 2024.

[ref25] Liu F.; Kong Y. Zoib: an R package for Bayesian inference for beta regression and zero/one inflated beta regression. R Journal 2015, 7 (2), 34–51. 10.32614/RJ-2015-019.

[ref26] R: A language and environment for statistical computing; R Foundation for Statistical Computing: Vienna, Austria, 2024. https://www.R-project.org/.

[ref27] NOWData - NOAA Online Weather Data. https://www.weather.gov/wrh/Climate?wfo=aly (accessed April 1, 2024).

[ref28] HYDROMET Data System. Bozeman Montana Weather Station 6W. https://www.usbr.gov/gp/hydromet/ (accessed July 31, 2024).

[ref29] Stein E. D.; Jerde C. L.; Allan E. A.; Sepulveda A. J.; Abbott C. L.; Baerwald M. R.; Darling J.; Goodwin K. D.; Meyer R. S.; Timmers M. A.; Thielen P. M. Critical considerations for communicating environmental DNA science. Environ. DNA 2024, 6 (1), e472 10.1002/edn3.472. PMC11110536 38784600

[ref30] Jensen M. R.; Sigsgaard E. E.; Ávila M. d. P.; Agersnap S.; Brenner-Larsen W.; Sengupta M. E.; Xing Y.; Krag M. A.; Knudsen S. W.; Carl H.; et al. Short-term temporal variation of coastal marine eDNA. Environ. DNA 2022, 4 (4), 747–762. 10.1002/edn3.285.

[ref31] Thalinger B.; Kirschner D.; Pütz Y.; Moritz C.; Schwarzenberger R.; Wanzenböck J.; Traugott M. Lateral and longitudinal fish environmental DNA distribution in dynamic riverine habitats. Environ. DNA 2021, 3 (1), 305–318. 10.1002/edn3.171.

[ref32] Morrison M. K.; Lacoursière-Roussel A.; Wood Z. T.; Trudel M.; Gagné N.; LeBlanc F.; Samways K.; Kinnison M. T.; Pavey S. A. Including environmental covariates clarifies the relationship between endangered Atlantic salmon (*Salmo salar*) abundance and environmental DNA. Environ. DNA 2023, 5 (5), 987–1003. 10.1002/edn3.424.

[ref33] Shogren A. J.; Tank J.; Andruszkiewicz E.; Olds B.; Mahon A.; Jerde C.; Bolster D. Controls on eDNA movement in streams: transport, retention, and resuspension. Sci. Rep. 2017, 7, 5065 10.1038/s41598-017-05223-1. 28698557 PMC5506058

[ref34] Turner C. R.; Uy K. L.; Everhart R. C. Fish environmental DNA is more concentrated in aquatic sediments than surface water. Biol. Conserv. 2015, 183, 93–102. 10.1016/j.biocon.2014.11.017.

[ref35] Mathieu C.; Hermans S. M.; Lear G.; Buckley T. R.; Lee K. C.; Buckley H. L. A systematic review of sources of variability and uncertainty in eDNA data for environmental monitoring. Front. Ecol. Evol. 2020, 8, 135 10.3389/fevo.2020.00135.

[ref36] Jo T. S. Utilizing the state of environmental DNA (eDNA) to incorporate time-scale information into eDNA analysis. Proc. R. Soc. B 2023, 290 (1999), 20230979 10.1098/rspb.2023.0979. PMC10229230 37253423

[ref37] Truelove N. K.; Patin N. V.; Min M.; Pitz K. J.; Preston C. M.; Yamahara K. M.; Zhang Y.; Raanan B. Y.; Kieft B.; Hobson B.; et al. Expanding the temporal and spatial scales of environmental DNA research with autonomous sampling. Environ. DNA 2022, 4 (4), 972–984. 10.1002/edn3.299.

[ref38] Preston C.; Yamahara K.; Pargett D.; Weinstock C.; Birch J.; Roman B.; Jensen S.; Connon B.; Jenkins R.; Ryan J.; et al. Autonomous eDNA collection using an uncrewed surface vessel over a 4200-km transect of the eastern Pacific Ocean. Environ. DNA 2023, 6 (1), e468 10.1002/edn3.468.

[ref39] Yamahara K. M.; Preston C. M.; Birch J.; Walz K.; Marin R. III; Jensen S.; Pargett D.; Roman B.; Ussler W. III; Zhang Y.; et al. *In situ* autonomous acquisition and preservation of marine environmental DNA using an autonomous underwater vehicle. Front. Mar. Sci. 2019, 6, 373 10.3389/fmars.2019.00373.

[ref40] USEPA. Definition and procedure for the determination of the method detection limit, revision 2; EPA 821-R-16–006; U.S. Environmental Protection Agency, 2016. https://www.epa.gov/sites/default/files/2016-12/documents/mdl-procedure_rev2_12-13-2016.pdf.

[ref41] Hendricks A.; Mackie C. M.; Luy E.; Sonnichsen C.; Smith J.; Grundke I.; Tavasoli M.; Furlong A.; Beiko R. G.; LaRoche J.; Sieben V. Compact and automated eDNA sampler for in situ monitoring of marine environments. Sci. Rep. 2023, 13 (1), 5210 10.1038/s41598-023-32310-3. 36997631 PMC10063616

